# Ambiguous subdural collection

**DOI:** 10.1002/ccr3.782

**Published:** 2016-12-26

**Authors:** Jonathan Nakhla, Niketh Bhashyam, Reza Yassari

**Affiliations:** ^1^ Department of Neurological SurgeryMontefiore Medical Center/Albert Einstein College of MedicineBronxNew YorkUSA

**Keywords:** Metastasis, prostate cancer, subdural mass, trauma, worsening mental status

## Abstract

Prostate cancer metastasis to the dura is a rare occurrence. Metastasis to the dura can present as signs and symptoms of worsening mental status or neurological deficit. Therefore, malignant metastasis should be considered in a patient presenting with history of prostate cancer and worsening metal status without evidence of trauma.

## Case Presentation

**Figure 1:**
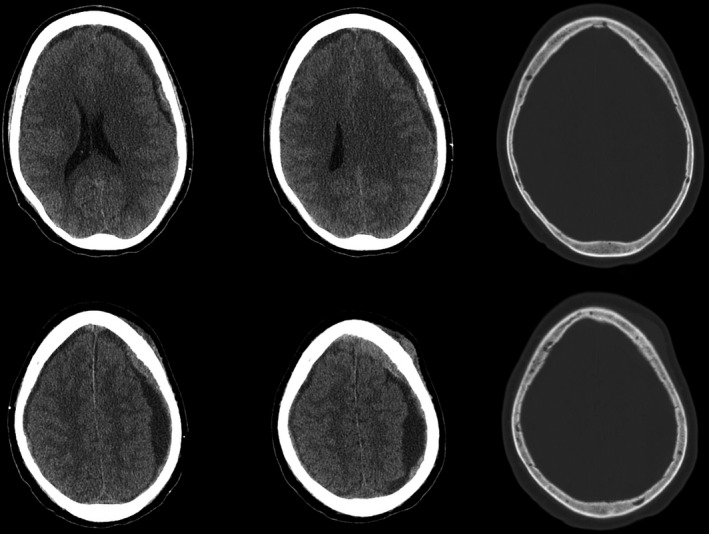
CT axial images of the head demonstrating left greater than right acute on chronic subdural hematomas with midline shift and mass effect without evidence of fracture or bony abnormalities.

**Figure 2:**
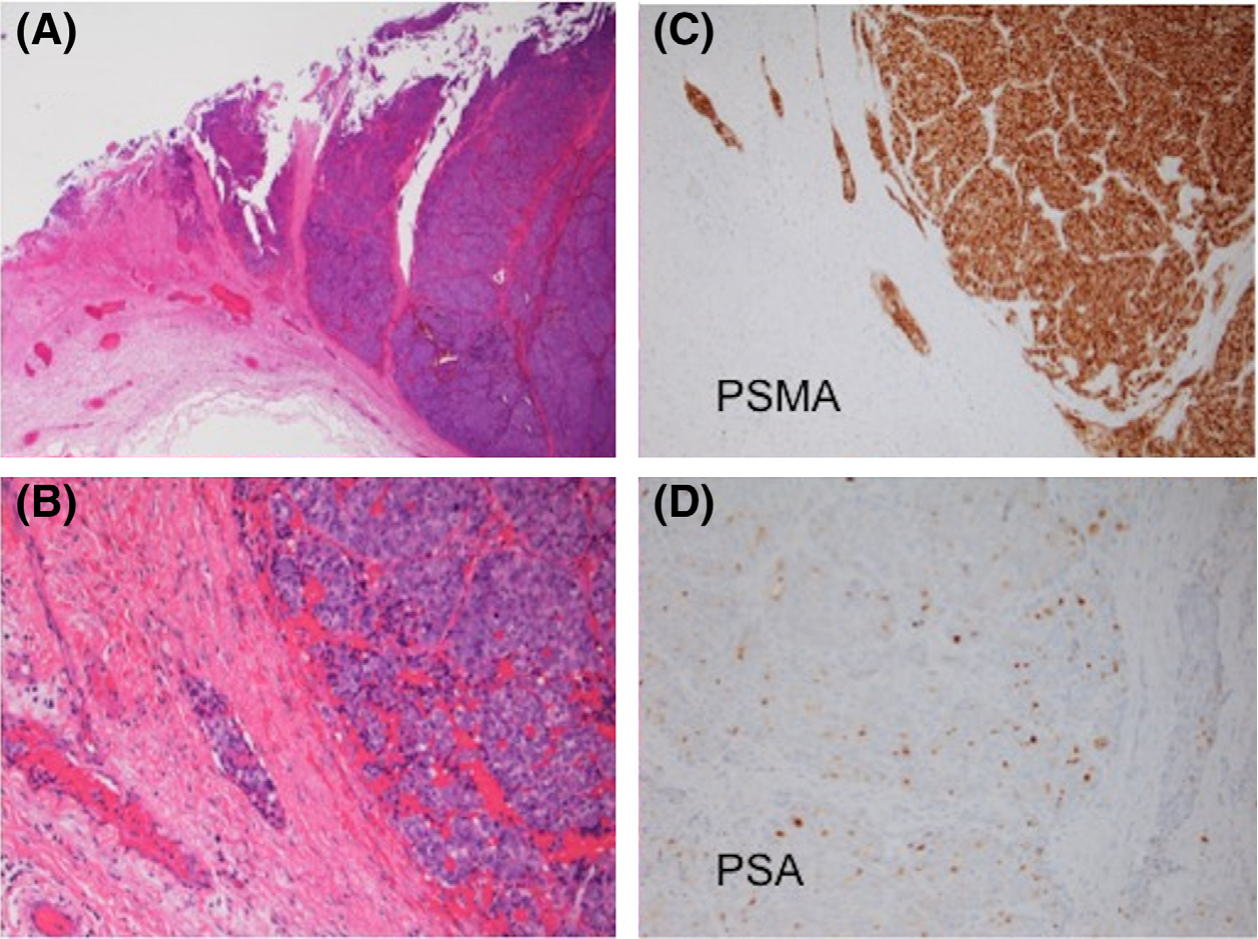
(A) Low‐power view: Cellular neoplasm forming nests invading the dura. (B) High‐power view: Large cells with moderate pink cytoplasm, vesicular nuclei and prominent nucleoli. Immunohistochemical stains for PSA (C) and PMSA (D) revealed a diagnosis is metastatic prostate carcinoma.

**Figure 3:**
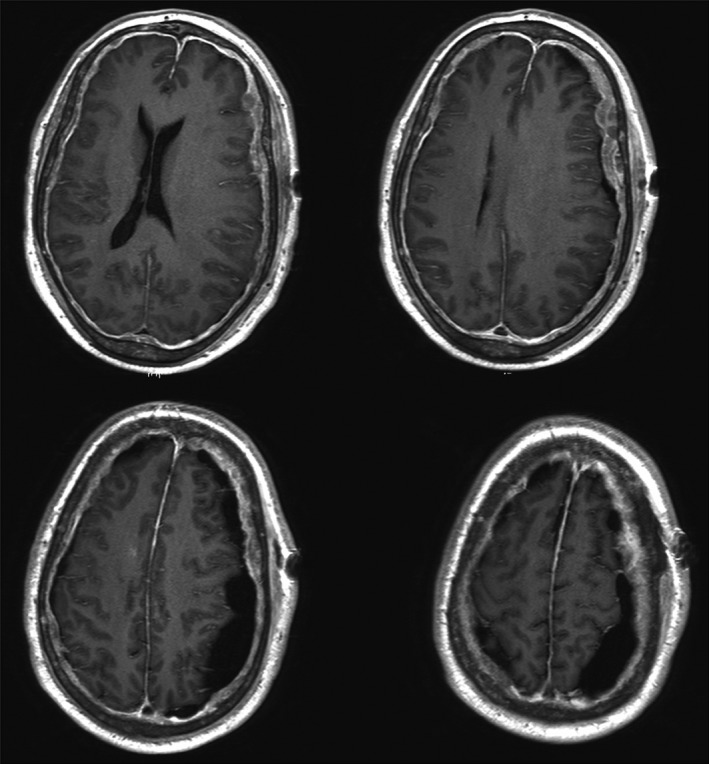
MRI of the brain with contrast reveals abnormal enhancement and thickening of the dura with resultant mass effect and midline shift.

A 61‐year‐old man with prostate cancer, presented with worsening mental status, without evidence of trauma. On examination, the patient was oriented to only person and place, without pronator drift, and was found to have subdural collections (Fig. 1). He was taken to the operating room for an evacuation where he was found to have a solid mass instead of fluid (Fig. 2). Postoperatively, on the contrary, MRI revealed the subdural mass with subsequent mass effect and shift (Fig. 3). Hence, the patient has expired.

## Discussion

Metastatic prostate cancer to the dura is a rare occurrence. In a review of 6282 patients, prostate cancer metastasized to the dura in 0.45% of patients 1. Lawton et al. reported that the mean survival of patients with metastatic prostate dural disease was 6.7 months 2.

## Conclusion

Subdural metastasis should be considered in the absence of trauma and in the presence of systemic malignancy.

## Authorship

JN: Neurological Surgery Resident – Main writer of this article. NB: Medical Student – Helped in the writing of this article. RY: Associate Professor of Neurosurgery; Director of Montefiore Spine Center, Surgical Services – Helped in the writing and editing of this article.

## Conflict of Interest

None declared.

## References

[1] Tremont‐Lukats, I. W. , G. Bobustuc , G. K. Lagos , K. Lolas , A. P. Kyritsis , and V. K. Puduvalli . 2003 Brain metastasis from prostate carcinoma: The M. D. Anderson Cancer Center experience.. Cancer 98:363–368. 12872358 10.1002/cncr.11522

[2] Lawton, A. , G. Sudakoff , L. C. Dezelan , and N. Davis . 2010 Presentation, treatment, and outcomes of dural metastases in men with metastatic castrate‐resistant prostate cancer: a case series. J. Palliat. Med. 13:1125–1129. 20836637 10.1089/jpm.2009.0416 PMC2964359

